# Can we improve recruitment to trials and informed consent by improving participant information sheets? - A nested RCT

**DOI:** 10.1186/1745-6215-12-S1-A123

**Published:** 2011-12-13

**Authors:** Peter Knapp, Natasha Mitchell, David K Raynor, Jonathan Silcock, Brian Parkinson, Janet Holt, Yvonne Birks, Simon Gilbody

**Affiliations:** 1 Department of Health Sciences, University of York, York, UK; 2 School of Healthcare, University of Leeds, Leeds, UK; 3 School of Pharmacy, University of Bradford, Bradford, UK; 4 Making Sense Design, Sheffield, UK

## Objectives

• Develop a more readable participant information sheet (PIS) for an ongoing trial (CASPER) through re-writing, graphic design and user testing; and

• Assess the impact of the enhanced sheet on trial recruitment and informed consent.

## Methods

CASPER is a UK NIHR-funded trial of Collaborative Care to prevent depression in adults aged 75+. During the CASPER set-up phase we produced an information sheet compliant with National Research Ethics Service (NRES) guidance (the ‘standard sheet’). We decided to try to enhance recruitment by improving the PIS (the ‘enhanced sheet’). The nested study comprises: first, development and testing of an ‘enhanced sheet’; second, comparison of the ‘standard’ and ‘enhanced’ versions in a nested RCT.

The comprehensibility of the NRES-approved standard sheet was assessed by asking cohorts of 10 older adults to take part in individual user testing interviews. After reading the sheet, participants were asked to find answers in the sheet to 18 factual questions and show their understanding of found information. The sheet was then re-written, re-structured and re-designed, drawing on graphics and writing expertise and the user testing data. The ‘enhanced sheet’ was user-tested on further cohorts of 10 people and amended according to the data obtained.

In a nested RCT, potential CASPER participants will be posted either the ‘standard’ or ‘enhanced’ sheet, to assess effects on interest in participation and recruitment. Responders will be asked to complete an abbreviated version of the Joffe Quality of Informed Consent measure [1]. We will also conduct 2 focus groups with participants to explore the role of the sheet in their decision to participate (or not).

## Results

Despite it being approved by NRES, testing of the standard CASPER trial sheet revealed limitations in document organisation and writing, resulting in difficulty understanding such issues as trial benefits, sources of patient data and trial withdrawal. Revision included the use of lay language and short sentences; new font, layout and sub-headings; document re-organisation including adding a contents list and summary section. Testing of the ‘enhanced sheet’ on 30 people showed significant improvements in finding and understanding of information.

During 2011-12 we will assess the impact of the two versions of the sheet on recruitment and consent in our trial of older people with low severity depression.

## Conclusions

User testing, expert re-writing and graphic design produced a more readable trial information sheet. To our knowledge this will be the first randomised evaluation of an enhanced trial sheet remaining NRES-compliant. These results will interest others concerned with the improvement of information for trial participants.
